# Influence of age range of dairy Gir cows on the composition of colostrum and milk, and on calves’ passive immunity transfer

**DOI:** 10.1590/1984-3143-AR2025-0133

**Published:** 2026-06-15

**Authors:** João Vitor Menezes Lopes, Maria Claudia Pereda Francischini, Beatriz Melo Justo, Viviani Gomes, Camila Infantosi Vannucchi

**Affiliations:** 1 Departamento de Reprodução Animal, Faculdade de Medicina Veterinária e Zootecnia, Universidade de São Paulo, São Paulo, SP, Brasil; 2 Departamento de Clínica Médica, Faculdade de Medicina Veterinária e Zootecnia, Universidade de São Paulo, São Paulo, SP, Brasil

**Keywords:** zebu, protein, immunoglobulin, albumin

## Abstract

Colostrogenesis and lactogenesis influenced by age require further understanding in Zebu herds, due to the longevity of breeding cows. Thus, we aimed to compare colostrum and milk physicochemical and proteinogram of Dairy Gir cows and calves’ passive immunity transfer by grouping 28 cows and their calves by maternal age: Young (n=6; aged 24-36 months), Adult (n=15; 37-91 months) and Senior (n=7; 96-137 months) groups. Colostrum and milk were collected at first milking (immediately after calving), transition milk (3^rd^ milking, after 24h of calving) and full milk (10^th^ milking, after 5 days of calving) for analysis of density, total solids, pH, immunoglobulin (IgG) and proteinogram (lactoferrin, albumin, heavy and light chain IgG, β-lactoglobulin and α-lactalbumin). Calves’ blood was collected after 3 days of birth to evaluate serum total solids, total protein, liver profile, light and heavy chain IgG. Data were statistically analyzed by LSD Test at P<0.05. Young Group had higher (*P*<0.05) colostrum total solid and density compared to Senior Group. Transition milk of young cows had lower (*P*<0.05) pH than seniors. Calves of senior cows presented higher (*P*<0.05) serum albumin than young and adult. Young cows had IgG decrease from colostrum to transition milk, whereas adult cows presented fall (*P*<0.05) of IgG throughout milking. In conclusion, age of Dairy Gir cows does not affect colostrum and milk immunological quality, nor calves’ immunity transfer. However, it determines protein composition and dynamic changes, especially in young and senior cows.

## Introduction

Zebu breeds are dual-purpose cattle with both quantitative and qualitative excellence for meat and milk. They are known for higher milk solids yield, carcasses with satisfactory marbling and, above all, resistance to climate as well as endo- and ectoparasites. The key factor driving the high demand for Zebu cattle is the efficiency of genetic improvement programs, surpassing the genetic quality of their counterparts in India (Verneque et al., 2000). The Gir breed is perhaps the oldest Zebu breed in the world, highly valued for its temperament and genetic potential, particularly in crossbreeding (Santos, 2022). However, since they are generally maintained in extensive management systems, there is a notable scarcity of literature reporting reference parameters. Therefore, despite the hardiness and economically important traits of Zebu cattle, their specific characteristics, especially regarding colostrum and milk, have not been fully described, particularly for dairy Zebu breeds, such as the Gir.

Among selection criteria, the longevity of breeding females is considered one of the most desirable traits, leading to prolonged productivity (Silva et al., 2018). Currently, most Zebu herds consist of a larger number of breeding cows aged 10 to 15 years. Thus, the processes of colostrogenesis and lactogenesis, influenced by the age of Zebu females, require further understanding. Colostrum plays a vital role in providing immune defense mechanisms and supplying essential nutrients to neonates. It is particularly rich in proteins, fat, growth factors, and, most importantly, immunoglobulins (Brandon et al., 1971; Piccione et al., 2010). However, the protective characteristics and antimicrobial properties of colostrum are directly related to the natural exposure of cows to antigens from various pathogens (Hurley and Theil, 2011). In taurine cows, the concentration of immunoglobulins in colostrum reaches its peak between the second and third calving (Franklin et al., 2005; Silper et al., 2012). On the other hand, Zebu breeds are known to produce lower volumes of milk compared to taurine breeds. Consequently, the lower milk yield, combined with the later onset of sexual maturity in Zebu cows, may directly influence the composition and concentration of immunoglobulins in colostrum, thereby providing better immune support (Silper et al., 2012).

Despite lower production, milk from Zebu cows has great potential for human consumption, as milk from Zebu breeds such as Guzerá and Gir contains higher fat and lactose levels compared to European-origin cows (Vercesi et al., 2012). The exact composition of Zebu milk is not yet fully elucidated, and, in the case of the Dairy Gir cows, the effect of age on the constituent characteristics of colostrum and milk remains unknown. Despite the practical importance of Zebu colostrum, knowledge about the composition and characteristics of the colostrum and milk according to the age of the cow remains scarce. Additionally, most Zebu calves receive colostrum directly through maternal suckling, making it difficult to control the efficiency and quality of colostrum. This trait is primarily due to the superior maternal ability of Zebu cows, as they are more sensitive to milk letdown when stimulated by the presence of their offspring (Baruselli et al., 2007; Araújo, 2014). Therefore, the use of colostrum from ageing Gir cows may have a possible impact on calves’ passive immune transfer, possibly predisposing them to future infectious complications. On the other hand, we hypothesize that Gir cows are capable of producing colostrum of adequate immunological quality and milk of sufficient protein composition to ensure colostral passive immune transfer, regardless of the age group. Consequently, senior cows can remain productive within the herds for a longer duration throughout their lives.

Hence, the present study aims to evaluate and compare various aspects of the physicochemical composition, protein content, and immunoglobulin G concentrations in the colostrum and milk of Dairy Gir cows based on age group (young, adult, and senior cows) according to the official registration of the breed, regardless of parity or without accounting for the effect of parity. We also aim to verify and compare the efficiency of passive immunity transfer in calves according to the maternal age.

## Methods

A prospective and observational study was conducted in a single dairy Gir herd, following consent to the experimental protocol and in accordance with ethical guidelines for the use of animals in research. The conditions for animal use in this experiment are in accordance with the Ethics Committee on Animal Use (CEUA-FMVZ-USP), under protocol N^o^ 6462230418.

### Animals and experimental groups

The experiment was conducted in a Dairy Gir herd under sanitary and nutritional control, in southeastern Brazil (Latitude: 21°25'48.9"S, Longitude: 42°37'23.8"W) during the summer. All animals were kept under the same environmental conditions, grazing on *Brachiaria* spp. pastures, supplemented with a specific mineral salt formulation for pre-calving and fed a maize silage-based diet. Water was provided *ad libitum*.

A total of 28 Dairy Gir cows and 17 calves (Table 1), all clinically healthy with body condition scores between 3 and 4, were used. Exclusion factors included dystocia, stillbirth, twinning, mastitis, or alterations in the color, odor, and viscosity of the colostrum and milk postpartum. Unfortunately, 11 calves were born unsupervised and thus, they were discharged from the neonatal evaluation to avoid biased results. Dairy Gir cows were classified by age group according to the Brazilian Zebu Breeders Association (ABCZ, 2018) Regulations and assigned for the following experimental groups:

**Table 1:** Descriptive information of Gir cows enrolled in the Young (n=6), Adult (n=15) and Senior (n=7) groups.

**Group**	**Cow Age (yrs)**	**Parity**
**Young (24-36 months of age)**	36	Primiparous
	35	
	33	
	36	
	35	
	35	
**Adult (37 and 91 months of age)**	69	Primiparous
	54	Primiparous
	59	Primiparous
	73	Primiparous
	51	Primiparous
	60	Primiparous
	65	Primiparous
	91	Pluriparous
	57	Pluriparous
	38	Pluriparous
	38	Pluriparous
	52	Pluriparous
	59	Pluriparous
	45	Pluriparous
	41	Pluriparous
**Senior (96-137 months of age)**	127	Pluriparous
	127	
	121	
	120	
	113	

**Young Group** (n = 6 primiparous cows and 5 calves): Gir cows aged between 24 and 36 months.

**Adult Group** (n = 15 cows – 9 multiparous and 6 primiparous cows, and 8 calves): Gir cows aged between 37 and 91 months.

**Senior Group** (n = 7 multiparous cows and 4 calves): Gir cows aged between 96 and 137 months.

To determine the ideal number of animals in each experimental group for statistical relevance, the power analysis was performed using the SAS Power and Sample Size (PSS) application (SAS Institute Inc., Cary, NC, USA), employing means and standard errors obtained from a previous pilot experiment, employing the same cows of the main experiment. Experimental variables were selected (milk density, lactoferrin concentration, and albumin in milk whey), demonstrating a power test of 0.99. Considering an acceptable statistical power greater than 0.80, the number of animals used in this experiment ensures the scientific value of the findings.

### Milk and colostrum evaluations

After cleaning and disinfecting the cows' teats, colostrum or milk samples were manually collected at the first milking (immediately after calving), the third milking (after 24 hours – named transition milk), and the tenth milking (after 5 days), considering a regimen of two milkings per day. The samples were aliquoted into 50 mL tubes and stored at -20ºC until processing.

#### Density, total solids concentration, and pH of colostrum and milk

Immediately after milking, the samples were evaluated for appearance, viscosity, odor, and color. If acceptable, 250 mL of the sample were deposited in a graduated cylinder (between 20 and 25ºC) and the milk density was evaluated using a colostrometer (lactodensimeter), which determines immunoglobulin concentrations on a scale from 50 to 140 mg/mL (high quality), between 20 to 50 mg/mL (moderate quality), and below 20 mg/mL (low quality), according to Quigley (1998).

To measure total solids concentration, 25 µL of the sample were deposited on the prism of an optical Brix refractometer, with a scale of 0-32%. For bovine colostrum, high quality was considered when the total solids percentage exceeded 21%, indicating >50 g of immunoglobulins/L (Quigley et al., 2013).

The pH of colostrum, transition milk, and milk was measured with a digital pHmeter Sartorius PB-11-P11.1 Basic with Sartorius PY-P11-2S electrode, after calibration to compensate for electrode variation using pH 7, pH 4 and pH 10 buffers. Samples measurement was made by immersing the electrode into each sample, taking care to clean the electrode between each measurement with distilled water. The readability of the pH meter was 0.01, with an accuracy of ± 0.01.

#### Milk serum proteinogram

To obtain whey serum, milk and colostrum samples were coagulated by adding 100 µL of rennet to 5 mL of each sample, adhering to the manufacturer's recommended ratio (7 mL of rennet per 10 L of milk). The mixtures were incubated in a water bath at 37°C for 20 minutes, followed by centrifugation at 16,000 x g for 20 minutes. The resulting milk serum was stored in 1 mL microtubes at -20°C until further processing (Silper et al., 2012; Deelen et al., 2014).

The protein fractions present in the milk whey were analyzed using polyacrylamide gel electrophoresis (SDS-PAGE, Bolt™ gradient gel 4 to 12%, Bis-Tris, 1.0 mm, Mini Protein Gel, Thermo Fisher), employing a vertical Bis-Tris gel electrophoresis system with SDS MES buffer, following the modified Laemmli (1970) technique.

To generate a standard curve for protein molecular weight and known concentrations, bovine serum albumin (BSA) was used (Bovine Serum Albumin – SDS-PAGE, Sigma-Aldrich). Different solutions were prepared with varying BSA concentrations to obtain the standard curve. A specific concentration of 0.25 mg/mL BSA was used as the concentration standard for all analyses.

Considering the total protein concentration in each sample, the milk whey was diluted 1:10 in MilliQ® water for samples with values exceeding 5.0 g/dL. Subsequently, all samples were diluted to reach a concentration of 0.6 mg/mL. The samples were then denatured by adding 1:1 sample buffer (containing sodium dodecyl sulfate and β-mercaptoethanol), homogenized at 100ºC (AccuBlock™ Digital Dry Bath, Labnet®) for 10 minutes.

For each gel run, 15 µL of the sample, 5 µL of the molecular weight standard (PageRuler™ Plus Prestained Protein Ladder, 10 to 250 kDa), and the protein concentration standard (BSA, 0.25 mg/mL) were loaded onto the gel. The gel plates were placed in the Mini Gel Tank – Invitrogen®, and the Bolt™ MES SDS Running Buffer solution was added *as per* the manufacturer’s specifications. The plates were subjected to an electric current of 180 volts until the run was completed, taking approximately 1 hour. After the run and protein fraction separation, the gel was removed from the plate and stained with 0.2% Coomassie blue solution for 10 minutes in a horizontal shaker. The excess dye was then removed, and the gel was placed in a destaining solution (acetic acid and methanol) in a horizontal shaker until the bands were clearly visible.

**Figure 1:**
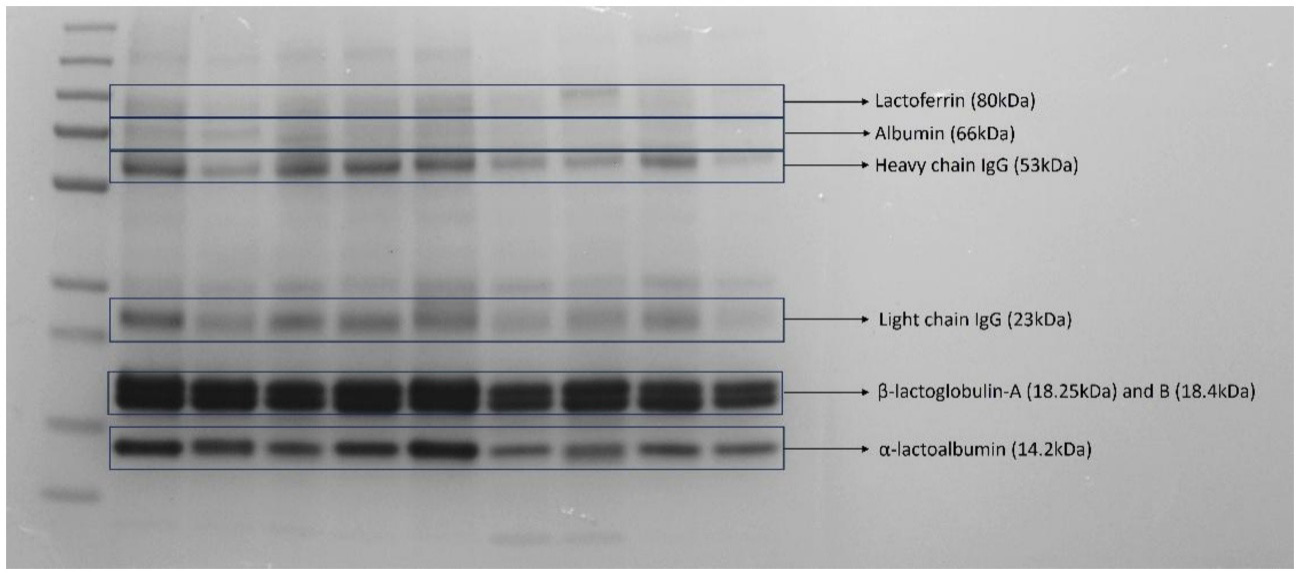
Milk serum proteinogram through SDS-PAGE electrophoresis gel showing the target protein bands and their respective molecular weights.

To capture photos and images of the gels, the MultiDoc-It Digital Imaging System UVP was used, revealing protein bands of different molecular weights. Due to the use of heat and the presence of denaturants (β-mercaptoethanol), disulfide bonds were broken, separating the heavy and light chains of immunoglobulin G. In this study, the following proteins were analyzed: lactoferrin (80 kDa), albumin (66 kDa), heavy chain immunoglobulin G (53 kDa), light chain immunoglobulin G (23 kDa), β-lactoglobulin A (18.25 kDa) and B (18.4 kDa), and α-lactalbumin (14.2 kDa) (Figure 1). The calculation of the concentration of each protein in the milk whey was performed by considering the relative percentage of the protein with the highest and lowest absolute concentration in the sample.

#### Milk serum immunoglobulin evaluation

Colostrum, transition milk, and milk samples were initially centrifuged for 30 minutes at 18,000 Xg to separate the serum from the fat fraction and casein pellet (Reber et al., 2008). The quantification of IgG was performed using sandwich ELISA immunofluorescence, with rabbit anti-bovine IgG antibody (capture antibody, B5645; Sigma, St. Louis, MO) diluted at a ratio of 1:400 in sodium carbonate buffer. Immulon 4HBX plates (Thermo Corp., Milford, MA) were coated and incubated at 4-8 °C overnight. The plates were then washed three times using phosphate-buffered saline containing 0.5% Tween 20. For the assay, milk whey samples were diluted 1:1,000,000 and 1:10,000,000. Samples were added in duplicate to wells and incubated for 1 hour at 20-26 °C. After incubation, the plates were washed three times. Bound or conjugated IgG was detected using rabbit anti-bovine IgG conjugated with horseradish peroxidase (detection antibody, A5295; Sigma, St. Louis, MO), diluted 1:1000, and incubated for 30 minutes (Reber et al., 2008). Sequentially, the plates were washed three times again. The concentration of bound detection antibody was measured using the substrate 2,2'-azino-bis (3-ethylbenzthiazoline-6-sulfonic acid; ABTS, A-9941; Sigma, St. Louis, MO), containing 20 μL of 30% hydrogen peroxide in 11 mL ABTS (pre-conditioned at room temperature for 15 minutes) and incubated for 30 minutes. Color signal reading was performed using a plate reader with a 405 nm filter, utilizing a standard curve of immunoglobulin with serial dilutions of bovine γ-globulin (I5506; Sigma, St. Louis, MO) in the range of 50 to 0.39 ng/mL (Reber et al., 2008).

### Neonatal evaluation

The calves were bottle fed colostrum from their own dams within maximum 8 hours after birth, with an average time between 4 and 6 hours. Colostrum volume was stablished to be 10% of calves body weight. To assess the efficiency of the full period of passive immunity transfer of the colostrum and transition milk, blood samples were collected from the calves on day 3 after birth via jugular venipuncture and deposited into tubes without anticoagulant. After centrifugation at 14,000 x g for 10 minutes, the serum obtained was stored at -20ºC until processing.

Samples of 50 µL of blood serum were placed on the optical prism of a Brix refractometer, and the value obtained, in a gradual scale, was expressed in g/dL of serum total solids (Deelen et al., 2014). Passive immunity transfer was considered effective for values above 5.5 g/dL of serum total solids concentration, while results between 5.0 and 5.4 g/dL indicated moderate passive immunity transfer. Failure of passive immunity transfer was defined for values below 5.0 g/dL (Quigley et al., 2013; Deelen et al., 2014).

In the calves’ blood serum, the concentrations of total protein, gamma-glutamyl transferase (GGT), aspartate aminotransferase (AST), albumin, direct and indirect bilirubin were determined using an automatic biochemical analyzer (Labtest® brand, Labmax 240 model). Commercial kits were used: Labtest® for total protein and albumin (Ref. Labtest Total Proteins 99 and Albumin 19) and BioSystems® for the enzymes (Ref. BioSystems GGT 11520, AST 11561 and total and direct bilirubin 11555).

For the analysis of the light and heavy chain Ig G fractions in the blood serum of calves, the SDS-PAGE polyacrylamide gel electrophoresis technique was employed, as previously described. Additionally, the total concentration of Ig G in the blood serum of the calves was determined by sandwich ELISA immunofluorescence, following the protocol previously described for milk whey. However, it is important to note that the neonatal blood serum samples were diluted at 1:1,000,000 for proper processing.

### Statistical analysis

The experimental design comprised three experimental groups evaluated across three distinct time points, classified as a Two-Way Mixed-Design ANOVA (or a Split-Plot Design).

The data obtained were analyzed using the SAS System for Windows 9.3. Through the Guided Data Analysis application, the data were tested for the normality of residuals (normal distribution) and homogeneity of variances. In cases where these assumptions were not met, the data were transformed (log 10 for GGT, albumin, light-chain Ig G, and pH variables; and square root for heavy-chain Ig G and lactoferrin variables). Effects of experimental group, time of evaluation, and interaction between these factors were estimated by repeated measures ANOVA - SAS MIXED procedure (Table 2).

**Table 2:** Probability values for the interaction between the main effects of groups (Young *vs.* Adult *vs.* Senior) and time (colostrum *vs.* transition milk *vs.* milk days) on milk composition.

	Group	Time	Group x Time
α-lactalbumin (%)	0.25	0.009	0.99
β-lactoglobulin (%)	0.18	0.17	0.90
Heavy chain Ig G (%)	0.68	<0.0001	0.67
Light chain Ig G (%)	0.45	<0.0001	0.94
Albumin (%)	0.35	0.0003	0.95
Lactoferrin (%)	0.68	<0.0001	0.25
ELISA IgG (mg/dL)	0.55	<0.0001	0.88
Density (g/dL)	0.05	<0.0001	0.23
Total solid concentration (mg/mL)	0.23	<0.0001	0.18
pH	0.79	0.59	0.07

The LSD test was used to compare differences over time and between age groups (young *vs.* adult *vs.* senior). Pearson correlation was used to assess the relationships between the response variables of parametric data. The significance level was set at 5%. The results are reported as untransformed means ± SEM.

## Results

**Figure 2:**
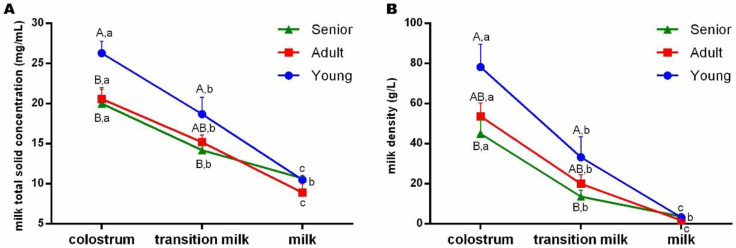
Mean and standard error (X±SE) of colostrum, transition milk and milk (A) total solid concentration and (B) density of Young, Adult and Senior Dairy Gir cows. ^a-c^ Different lowercase superscripts represent statistical difference (*P*<0.05) between milking samples within the same group; ^A-B^ Different uppercase superscripts represent statistical difference (*P*<0.05) between age related groups within the same milking sample.

Regardless of the age group, all cows exhibited a decreasing concentration (*P*<0.05) of milk total solid (Figure 2A) and density (Figure 2B) throughout the milking samples (colostrum, transition milk and milk). However, senior Gir cows kept solid concentration and density statistically constant (*P*>0.05) from the transition milk to milk, whereas young and adult cows had an additional decrease (*P*<0.05) until 5 days of milking (Figure 2). On the other hand, Young Group had higher (*P*<0.05) total solid concentration in the colostrum and, for the transition milk, it was higher (*P*<0.05) only compared to the Senior Group (Figure 2A). Furthermore, colostrum and transition milk of young Gir cows had higher (*P*<0.05) density comparing to senior cows (Figure 2B).

Across different milking samples (colostrum, transition milk, or milk) of the age groups, no significant difference (*P*>0.05) was observed for the pH (Table 3). However, transition milk (24 h) of young Gir cows (6.18±0.1) had lower (*P*<0.05) pH than of senior cows (6.44±0.12), but not different (*P*>0.05) from the Adult Group (6.29±0.04).

**Table 3:** Mean and standard error (X±SE) of colostrum transition milk and milk pH of different age-related (Young, Adult and Senior) Dairy Gir cows.

	**Young Group**	**Adult Group**	**Senior Group**
Colostrum	6.47 ± 0.12	6.32 ± 0.03	6.27 ± 0.05
Transition milk	6.18 ± 0.10^a^	6.29 ± 0.04ab	6.44 ± 0.12^b^
Milk	6.32 ± 0.01	6.33 ± 0.02	6.34 ± 0.03

^a-b^Values with different superscripts in the same line differ significantly between experimental groups (P< 0.05).

**Figure 3:**
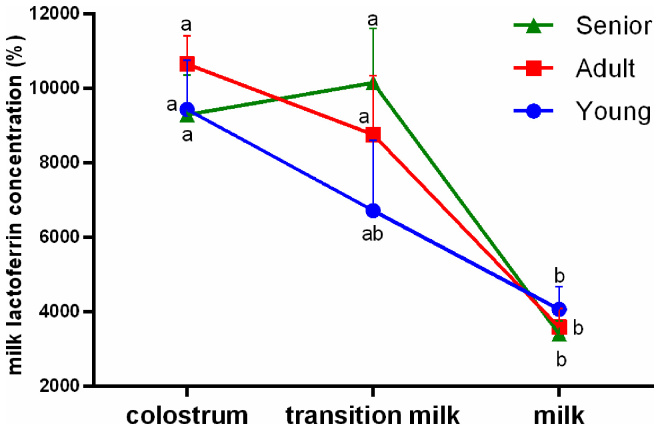
Mean and standard error (X±SE) of colostrum, transition milk and milk lactoferrin of Young, Adult and Senior Dairy Gir cows. ^a,b^Different lowercase superscripts represent statistical difference (*P*<0.05) between milking samples within the same group.

There was no significant difference (*P*>0.05) in milk proteinogram β-lactoglobulin throughout milking samples of the different age-related groups. However, senior Gir cows had higher (*P*<0.05) milk (5-day sampling) α-lactalbumin (10,666.31±1,410.77%) compared to Young Group (8,346.91±353.72%). In addition, young Gir cows had a progressive decrease (*P*<0.05) of lactoferrin during the transition from colostrum to milk, whereas the Adult and Senior groups presented an acute decrease (*P*<0.05) of lactoferrin from transition milk to milk (Figure 3).

**Figure 4:**
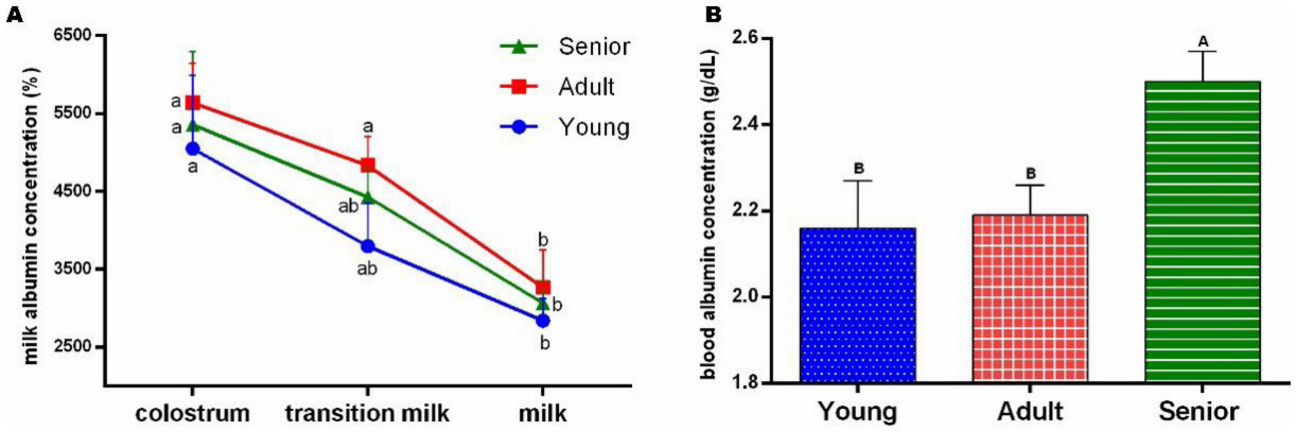
Mean and standard error (X±SE) of colostrum, transition milk and milk (A) whey albumin and (B) blood calf albumin concentration of Young, Adult and Senior Dairy Gir cows. ^a,b^ Different lowercase superscripts represent statistical difference (*P*<0.05) between milking samples within the same group; ^A-B^ Different uppercase superscripts represent statistical difference (*P*<0.05) between age related groups.

Concerning the milk whey concentration of albumin, we observed a decreasing (*P*<0.05) profile in all cows, nevertheless, adult Gir cows had a sharp fall (*P*<0.05) from transition milk to milk (Figure 4A). Interestingly, such milk albumin profile reflected differently in calves’ blood albumin concentration, i.e., calves born from senior Gir cows presented higher (*P*<0.05) blood albumin level compared to Young and Adult groups (Figure 4B).

**Figure 5:**
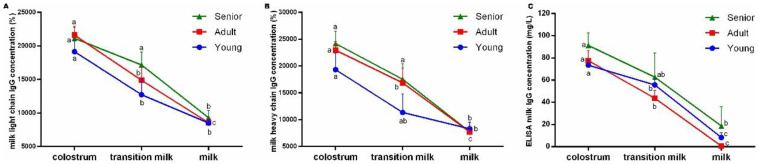
Mean and standard error (X±SE) of colostrum, transition milk and milk whey (A) light chain immunoglobulin G and (B) heavy chain immunoglobulin G concentration and (C) sandwich ELISA immunofluorescence IgG concentration of Young, Adult and Senior Dairy Gir cows. ^a,c^ Different lowercase superscripts represent statistical difference (*P*<0.05) between milking samples within the same group.

Irrespective of the milking sample (colostrum, milk transition and milk), whey IgG concentrations did not differ (*P*>0.05) among the experimental age related-groups for the proteinogram detection, while a physiological decrease (*P*<0.05) from colostrum to milk samples was observed (Figure 5A and 5B). Nonetheless, such diminution occurred differently compared to each age-related group. Young Gir cows had an acute light chain IgG decrease (*P*<0.05) from colostrum to milk transition, whereas adult cows presented a significant fall (*P*<0.05) of both light and heavy chain IgG throughout milk sampling (Figure 5A and 5B). It is interesting to note that transition milk of senior Gir cows remained unchanged (*P*>0.05) for the light chain IgG since first milking, presenting a significant decrease (*P*<0.05) only after 5 days of sampling (Figure 5A). For the specific ELISA IgG detection (Figure 5C), milk whey IgG profile differed throughout the milking process differently for each experimental group, i.e., while young and adult Gir cows had a significant decrease (*P*<0.05) from colostrum to milk, IgG concentration lowered significantly (*P*<0.05) only after 5 days (milk) in senior cows (Figure 5C).

On the other hand, it is worth noting that no difference (*P*>0.05) of ELISA-detected blood IgG was observed for calves of different age-related groups (Young, Adult and Senior; Table 4). Additionally, comparing the calves’ variables across the different maternal age groups of Gir cows, no statistical differences (*P*>0.05) were observed (Table 4).

**Table 4:** Mean and standard error (X±SE) of blood total solids concentration, liver profile and ELISA-detected blood IgG of calves of different age-related (Young, Adult and Senior) Dairy Gir cows.

	**Young**	**Adult**	**Senior**
Total solid concentration (mg/mL)	9.76 ± 0.66	8.50 ± 0.32	9.25 ± 0.62
Total protein (g/dL)	6.50 ± 0.56	5.84 ± 0.35	6.25 ± 0.53
Aspartate aminotransferase (UI/L)	20.28 ± 4.18	17.21 ± 1.67	26.60 ± 4.54
Gamma-glutamyl transferase (UI/L)	706.34±240.06	524.08±172.43	400.17±128.85
Total bilirubin (UI/L)	0.43 ± 0.06	0.40 ± 0.05	0.72 ± 0.27
Direct bilirubin (UI/L)	0.22 ± 0.04	0.23 ± 0.02	0.27 ± 0.03
ELISA-detected IgG (mg/L)	45.75±7.76	45.12±7.28	64.12±11.68

For the Young Group, a significant positive correlation was found between milk whey IgG concentration and milk density (r=0.84; *P*<0.0001), total solids concentration (r=0.94; *P*<0.0001), and lactoferrin concentration (r=0.53; *P*=0.03). In the Adult Group, positive correlations were also observed between milk IgG concentration and milk density (r=0.83; *P*<0.0001), total solids concentration (r=0.81; *P*<0.0001) and lactoferrin concentration (r=0.43; *P*=0.01). Moreover, the concentration of milk whey IgG negatively correlated with α-lactalbumin concentration (r=-0.47; *P*=0.007). For the Senior Group, a significant positive correlation was found between milk whey IgG concentration and total solids concentration (r=0.57; p=0.01). Additionally, whey lactoferrin concentration positively correlated with light-chain IgG concentration (r=0.85; *P*<0.0001), heavy-chain IgG concentration (r=0.81; *P*<0.0001), and only in the Senior Group, with whey albumin concentration (r=0.69; *P*=0.001).

For the analyses of neonatal calves born to Dairy Gir cows of different age groups, in the young and adult groups, a positive correlation was found between serum IgG concentration and total protein concentration (r=0.91; *P*=0.03 and r=0.93; *P*=0.0006, respectively). In the group of calves born to adult cows, a positive correlation was observed between blood serum IgG concentration and GGT (r=0.92; *P*=0.0009), and total solids (r=0.87; *P*=0.004). In the senior group, serum GGT concentration positively correlated with total solids concentration (r=0.98; *P*=0.01) and total protein concentration (r=0.96; *P*=0.03).

## Discussion

The present research describes physicochemical, protein, and immunological parameters of colostrum and milk of age-related Dairy Gir cows, as well as the efficiency of passive immunity transfer to their neonatal calves.

It was not possible to observe any influence of the age of Gir cows on milk whey immunoglobulin G concentration during the first 5 days of milking (colostrum, transition milk and milk). Although an increase in the colostrum immunoglobulin-G concentration has been described in Holstein cows with animal aging, this relationship could not be established for Dairy Gir cows in the condition herein. For the Holstein, younger cows have higher colostrum IgG concentrations compared to older cows, however such difference was related to the number of lactations, irrespective of dams age (Oyeniyi and Hunter, 1978; Pritchett et al., 1991). Nevertheless, despite the undetected influence of age-related group, we observed a different dynamic change in total IgG from first milking (colostrum) to 5-day milk according to the age of the cows. In the present research, senior Gir cows had a significant decrease in IgG concentration only after 5 days of milking, while young and adult cows presented an earlier whey IgG fall (yet at 24 hours of milking - transition milk). This result suggests a prolonged translocation of blood immunoglobulin G to the mammary gland in older Gir cows, a mechanism that deserves further clarification with a higher number of animals for this specific bovine sub-species. Due to the fact that calves of all age-related Gir cows had an even passive immune transfer, as to a similar blood IgG concentration, our results highlight the safety of using older Dairy Gir cows in breeding programs without compromising colostrum and milk quality. Moreover, in our conditions, senior Gir cows can be considered excellent colostrum bank donors, taking into account the prolonged IgG concentration during the first 5 days of milking, optimizing the volume of milk to be stored.

On the other hand, young Dairy Gir cows in the present experiment showed a higher concentration of total solids and density in colostrum and transition milk compared to senior cows. Despite the positive correlation between density and total solids with the colostrum and milk IgG concentrations in young cows, which could potentially indicate colostrum quality in our research, no statistical difference was found between age groups for milk whey IgG concentrations. It is important not only to note that we used a higher age range in each group, but also that the density and percentage of total solids in the sample includes also lipids (fat), carbohydrates, proteins, minerals, and vitamins. Particularly, the Gir breed is considered to have a high percentage of fat (triglycerides, diacylglycerides, saturated and polyunsaturated fatty acids) in the milk among Indian cattle breeds (Devi et al., 2025). Therefore, it can be inferred that these results may be due to the differential composition of other elements, such as phospholipids (which was beyond the scope of our study), and not necessarily related to immunoglobulin concentration in young Gir cows. In fact, colostrum quality regarding total solid concentrations is prolonged in Guzerá and Gir cows, with higher fat and protein values throughout lactation (Ribeiro et al., 2009; Araújo, 2014; Chaudhari et al., 2025). Thus, implementing colostrum banks from zebuine cows is extremely promising for dairy farms in general, particularly for more debilitated neonates or those with greater energy requirements. On the other hand, for such a purpose, future studies should be conducted to detail all solid-forming elements in the colostrum and milk of Gir cows, particularly concerning not only parity but also the dam´s age, and the development of tools to monitor colostrum quality in zebuine cows in addition to density and percentage of solid concentration.

In the present research, young Gir cows had lower α-lactalbumin production in the mammary gland (milk whey concentration) compared to senior ones, but there was no statistical difference with the Adult Group, possibly due to a higher age range in each group. The fact that young cows require greater energy expenditure for various physiological functions, such as growth and body development (Araújo, 2014), can be the one reason for the difference in milk production and composition. In fact, Hingonekar et al. (2025) showed that <900 days old Gir halfbreds have lower milk yield in first lactation than ≥1001 days old cows. With a significant biological role in milk production, α-lactalbumin has its production regulated by prolactin and serves as a precursor in the biosynthesis of lactose within the mammary gland parenchyma (Farrell et al., 2004; Bleck et al., 2009). We can thus assume that lactose content in the milk of younger Gir cows in this experiment can be lower compared to older cows, due to a parallel profile with α-lactalbumin concentration, an association that definitively deserves further investigation. Although lower lactose composition is an undesirable trait for consumption of milk for energetic purposes, the total milk content presented by young Gir cows herein still resemble the general milk production patterns in bovines. Thus, milk from young Gir cows is fully suitable for human consumption and may be recommended specifically for use by lactose intolerant individuals. Nonetheless, future studies on milk energy composition of dairy Gir cows should be conducted with a higher number of animals and considering parity, in order to truly determine the differences in lactose concentration according to the cows age range.

As a co-regulator of lactose synthesis in the epithelial cells of the mammary gland, α-lactalbumin also plays an important role in osmotic regulation and, coupled with albumin, are main factors in the formation of the aqueous part of milk (Farrell et al., 2004; Bleck et al., 2009). Therefore, genotypic variations in the production of α-lactalbumin and albumin may indicate the genetic potential for milk production volume (Brew, 2013). Although we have not determined milk volume yield across different age-ranged cows, the transition milk (24 h) pH in young Gir cows in this experiment was lower than seniors. According to Raimondo et al. (2009), lower milk pH is due to a higher concentration of total solids, which was also a result observed in the present research (young cows had higher density and total solids in colostrum and transition milk compared to older cows). Thus, we can assume that young Gir cows included herein do not exhibit complete dilution of transition milk compared to senior cows, supporting the notion of a greater milk dilution in cows with established milk production capacity. In other words, young Gir cows synthetize less milk α-lactalbumin and lactose, negatively impacting the total volume of milk produced by each cow, whereas older Gir cows stablish lactation volume more uniformly with aging. However, albumin content falls rapidly in adult cows from transition milk to 5-days milking in the present experiment, whereas a more uniform decrease was observed in senior cows, suggesting that milk yield can be counterbalanced by aging in the fifth day of milking.

Although no significant difference was observed for the majority of the serum variables examined in the calves herein, it is important to note that the albumin concentration differed according to maternal age, with calves from senior Gir cows exhibiting higher albuminemia compared to those from young and adult cows. Blood albumin concentration can be influenced by dietary protein availability and serves as an indicator of milk protein content, although changes in blood levels occur slowly due to the low synthesis and degradation rates (Belinskaia et al., 2021). Nevertheless, it can be suggested that the higher protein content in the milk of senior Gir cows in our conditions contributes to the increased serum albumin concentration in calves, thereby enhancing the blood transport of free fatty acids, amino acids, minerals, calcium, and hormones.

Despite the reported functional loss of the mammary gland parenchyma in older cows (Soares et al., 2009), leading to a direct reduction in productive capacity during lactations, the present experiment did not observe significant differences in colostrum and milk protein composition between adult and senior Dairy Gir cows. Adult and multiparous cows possess greater organic and digestive capacity, as well as better cardiorespiratory performance, making colostrogenesis, lactogenesis, and lactation establishment more efficient in crossbred taurine and zebu cows (Carvalho et al., 2001). Thus, our results highlight the safety of using older Dairy Gir cows in breeding programs without compromising colostrum and milk quality. This, in turn, allows for increased longevity of cows in herds, optimizing production per animal. Ultimately, better utilization of lactating cows makes Dairy Gir farming more viable and profitable from both a productive and commercial standpoint.

Lactoferrin is a multifunctional iron-binding glycoprotein with broad-spectrum antimicrobial activity and significant mediation of the immune response, playing a protective role in the mammary gland and gastrointestinal tract of neonates (Zimecki et al., 2005). In this experiment, the lactoferrin concentration profile during the initial days of lactation varied across age groups, showing a progressive decline in the transitional milk of young cows and an abrupt reduction in that of adult and senior cows. In fact, Tortadès et al. (2023) verified that 72.3% of lactoferrin concentration is conserved from colostrum to the transition milk (second milking) and that milk of multiparous cows has 34% more lactoferrin than primiparous. Thus, it is hypothesized that older Gir cows exhibit a faster inflammatory response in the mammary gland during milk formation on the fifth day of lactation. In fact, the Gir is one of the indigenous cattle breeds that has the greatest colostrum phagocytic activity, secretory immunoglobulin (IgG and IgA) levels, stronger anti-inflammatory responses, and lower pro-inflammatory cytokine levels (Kumawat et al., 2026). Although lactoferrin concentrations in colostrum are higher in dairy cows compared to beef cows, heifers (primiparous) have lower lactoferrin levels than multiparous cows, suggesting a lower incidence of mammary infections in the former (Tsuji et al., 1990). Additionally, milk lactoferrin concentration in young and senior Gir cows positively correlated with milk IgG concentration. Hence, in any case, the gradual reduction in lactoferrin concentration in the milk of young Gir cows facilitates iron intake by calves and potentially contributes to the development of neonatal intestinal defense mechanisms.

Although we prioritized the specific approach to the Gir categorization into specific age groups according to the breed association's official registration, a limitation of the present research is the naturally confounded effect of parity. Another important limitation of this study is the uneven number of cows in each experimental group and more importantly the low number of calves in the Senior Group, due to unsupervised calving. Therefore, future studies should be conducted using Gir cows of equal parity without taking into account a specific age category which, hence, could shed light on the interaction of age and parity in the specific Gir breed.

## Conclusion

In conclusion, the age of Dairy Gir cows does not affect the passive immunity transfer to their calves nor the immunological quality of colostrum and milk, although senior Gir cows take longer to show immunological decline in the transition from colostrum to milk for up to 5 days. In addition, colostrum and milk composition does have an age-related and overtime dynamic change with special reference to young Gir cows. However, future studies should focus on determining all solid-forming elements in the colostrum and milk of Gir cows with a larger number of animals in each age category in order to consistently attest the suggested events presented herein.

## Data Availability

The datasets generated during and/or analysed during the current study are available upon request.
